# Anti-Biofilm Activity of Combinations of Cinnamic Acid and Its Derivatives with Cloxacillin Against Methicillin-Resistant *Staphylococcus epidermidis*

**DOI:** 10.3390/cimb48030336

**Published:** 2026-03-23

**Authors:** Tomasz Zawiła, Denis Swolana, Marta Zawiła, Zuzanna Rzepka, Robert D. Wojtyczka

**Affiliations:** 1 Department of Microbiology, Faculty of Pharmaceutical Sciences in Sosnowiec, Medical University of Silesia in Katowice, Jagiellońska 4, 41-200 Sosnowiec, Poland; d200969@365.sum.edu.pl (T.Z.); dswolana@sum.edu.pl (D.S.); 2 Śląskie Laboratoria Analityczne, Wojska Polskiego 16h, 41-600 Świętochłowice, Poland; mmkwasniewska@gmail.com; 3 Department of Pharmaceutical Chemistry, Faculty of Pharmaceutical Sciences in Sosnowiec, Medical University of Silesia in Katowice, Jagiellońska 4, 41-200 Sosnowiec, Poland; zrzepka@sum.edu.pl

**Keywords:** antimicrobial activity, biofilm, cinnamic acid, *Staphylococcus epidermidis*

## Abstract

*Staphylococcus epidermidis* (*S. epidermidis*) poses a significant clinical challenge, particularly in the context of biofilm-associated infections, with increasing antibiotic resistance further complicating infection eradication. In the present study, the effects of cinnamic acid and its derivatives (ferulic acid, *p*-coumaric acid, and sinapic acid), alone and in combination with the β-lactam antibiotic cloxacillin, on biofilm formation by a single methicillin-resistant *S. epidermidis* (MRSE) clinical strain were explored. The expression of the biofilm-associated *icaADBC* operon genes and the *icaR* repressor gene was assessed using Real-Time PCR as an exploratory analysis under sub-minimal inhibitory concentrations (sub-MICs) of the tested compounds. Furthermore, confocal microscopy was used to qualitatively assess selected structural changes in the biofilm. Their occurrence was demonstrated depending on the fractional inhibitory concentration (FIC) levels used. The results revealed variable and nonlinear patterns of gene expression in response to the tested concentrations. Additionally, compound-dependent differences in anti-biofilm-related responses were observed. Overall, the findings provide insight into the potential influence of cinnamic acid derivatives combined with cloxacillin on biofilm-associated processes in *S. epidermidis*.

## 1. Introduction

*Staphylococcus epidermidis* (*S. epidermidis*) belongs to the group of coagulase-negative staphylococci (CoNS) colonizing human skin. Although less virulent than *Staphylococcus aureus* (*S. aureus*), *S. epidermidis* encodes multiple enzymes promoting adhesion, biofilm formation, survival, and immune modulation, particularly on medical devices [1]. The frequent isolation of this microorganism from clinical specimens necessitates the differentiation between contamination and a potentially life- or health-threatening infection, which requires significant labor, staff experience, and time [2]. The widespread use of synthetic medical devices has highlighted the clinical relevance of *S. epidermidis* as a biomaterial-associated pathogen, particularly due to its ability to adhere to foreign surfaces and form biofilm [3]. This competence, together with its ubiquity on human skin, contributes to its frequent recovery from infected devices, although it is not unique compared with other opportunistic pathogens [3]. Colonized medical devices (e.g., catheters, implants, prostheses) can serve as a source of recurrent bacteremia and may lead to sepsis and septic shock. Therefore, understanding the molecular aspects of biofilm formation appears crucial [2]. Biofilms are multicellular microbial communities embedded in an extracellular matrix of proteins, polysaccharides, and nucleic acids [4]. Cells within biofilms are protected against environmental stressors and show spatially heterogeneous metabolic activity, contributing to resistance to antimicrobial agents [5].

Polysaccharide intercellular adhesin (PIA), also known as poly-β-N-acetylglucosamine (PNAG), is a well-characterized component of staphylococcal biofilms that contributes to intercellular adhesion and structural stability. However, *S. epidermidis* strains may form biofilms in an *ica*-independent path.

Biofilm formation is a widespread trait among bacteria, including numerous staphylococcal species. Although *S. aureus* and *S. epidermidis* are the most common staphylococci associated with device-related biofilms, other coagulase-negative staphylococci are also capable of forming biofilms and may similarly cause biomaterial-associated infections [6]. Interestingly, this compound is not unique to staphylococci or Gram-positive bacteria; its presence has also been confirmed in phylogenetically distant bacteria such as *Escherichia coli* and *Pseudomonas fluorescens* [7]. Polysaccharide intercellular adhesin (PIA/PNAG) contributes to biofilm stability, though *ica*-independent biofilm formation also occurs [8]. The ability to synthesize PIA (PNAG) is determined by the presence of the *ica* genes located within the *icaADBC* operon. These genes are not part of the core staphylococcal genome and are variably present across species [9]. The synthesis of PNAG/PIA, a key component of biofilm, is regulated by the *icaADBC* operon and modulated by environmental and cellular factors, with the *icaR* gene acting as a global regulator [3].

Biofilms pose a significant threat to the health and lives of patients undergoing invasive procedures involving synthetic materials, such as in orthopedics. The structure of the biofilm necessitates an increase in antibiotic concentrations by up to 100–1000 times to effectively eradicate bacterial cells [3,10]. Another major concern is the increasing antibiotic resistance among staphylococci (e.g., methicillin-resistant *Staphylococcus epidermidis*—MRSE), which limits the therapeutic options by excluding β-lactam antibiotics, known for their high efficacy and safety profiles [11,12]. Consequently, there is an urgent need to search for new substances with antimicrobial activity. An interesting group of compounds exhibiting broad-spectrum activity (including both anti-planktonic and anti-biofilm effects) are cinnamic acid and its derivatives [13]. Cinnamic acid (CA) and its derivatives (*p*-coumaric acid (p-CA), ferulic acid (FA), and sinapic acid (SA)) are compounds widely distributed throughout the plant kingdom. These are natural carboxylic acids, characterized by the presence of an acrylic acid group substituted onto a phenyl ring [14].

**Figure 1:**
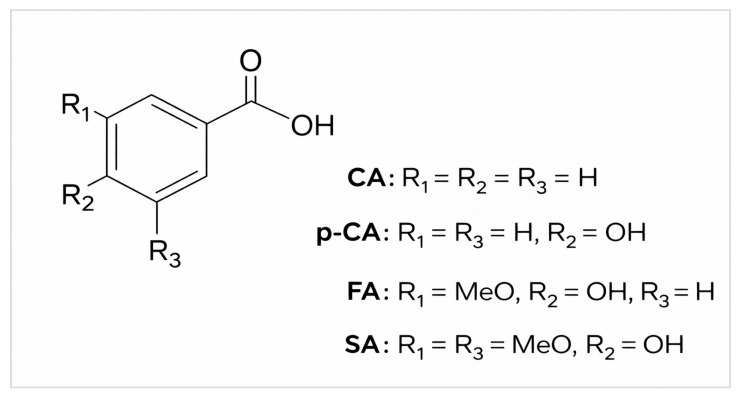
Structural formulas of cinnamic acid and its derivatives; CA—cinnamic acid; FA—ferulic acid; p-CA—*p*-coumaric acid; SA—sinapic acid.

CA exhibits a broad spectrum of pharmacological properties. This compound has found applications in the cosmetic industry, where its fragrance-related properties and its ability to protect against UV radiation are utilized [15]. Numerous studies conducted worldwide indicate that CA demonstrates not only antioxidant, neuroprotective, and anticancer activity [16], but also a wide range of antimicrobial properties. CA exhibits antibacterial [16,17,18,19], antifungal [20], and antiviral activity [21]. p-CA and FA are also characterized by a broad spectrum of activity against Gram-positive and Gram-negative bacteria, as well as fungi [16,22]. Both compounds also exhibit anti-inflammatory effects [23]. Moreover, FA demonstrates beneficial effects in cosmetology, including anti-aging activity related to UV exposure, as well as antioxidant and antimelanogenic activity [24]. In turn, p-CA also exhibits antioxidant, antidiabetic, and anticancer activity [15]. SA shows effects similar to those of the compounds mentioned above. These include antimicrobial [16], anti-inflammatory [25,26], antioxidant [25], and anticancer properties [27]. Moreover, they exhibit activity against non-fermentative bacilli. They are also active against yeast-like fungi and molds [16,28,29], and additionally demonstrate antimycobacterial activity [30]. The structural formulas of these acids are presented in Figure 1.

These acids are synthesized in plants via the Shikimate pathway, being derived from the amino acids L-Phe and L-Tyr. A sequence of hydroxylation and methylation reactions from either CA or p-CA leads to the derivatives caffeic acid, hydroxyferulic acid and SA. CA and p-CA serve as precursors of coumarins, while coumarylo-CoA along with malonyl-CoA act as precursors of flavonoids, stilbenes, and tannins. p-CA, FA and SA also serve as biosynthetic precursors of lignins and lignans [31].

The aim of this study was to evaluate the effect of CA, its derivatives, and the β-lactam antibiotic cloxacillin (CLX) at various concentrations on cell viability and biofilm biomass reduction of a methicillin-resistant *S. epidermidis* (MRSE) clinical strain. The effect of these substances on the molecular mechanisms underlying biofilm formation was also investigated. In particular, the study focused on analyzing the combined effect of these compounds on the expression of genes responsible for biofilm synthesis (*icaA*, *icaB*, *icaC*) and on assessing whether this combination affects the activity of the regulatory gene *icaR*. Simultaneously, biofilm formation capacity and bacterial cell count were assessed using confocal laser scanning microscopy at concentrations corresponding to and below the fractional inhibitory concentration (FIC).

## 2. Materials and Methods

### 2.1. Tested Strains

The clinical strains of *Staphylococcus epidermidis*, originating from the Microbial Collection of the Department and Division of Microbiology at the Medical University of Silesia, were selected for the study. These strains were originally isolated from vascular infections and demonstrated the highest biofilm-forming ability [13]. All experiments were performed using a single MRSE strain. The MIC values for CA, FA, p-CA, and SA for this strain were 4096 mg/L and for CLX—512 mg/L [30]. In Section 3.1.2, a methicillin-sensitive *S. epidermidis* (MSSE) clinical strain from the same collection was additionally used for comparison purposes. For this strain, all determined MIC values for the tested acids were 4096 mg/L. The species identification of the strains, with 99.99% probability, was performed using mass spectrometry (bioMerieux Vitec MS Prime^®^ system, Craponne, France).

### 2.2. Bacterial RNA Isolation

To the culture medium TSB with a 0.5% glucose supplement, a combination of the selected carboxylic acid (Aldrich Chemistry, Saint Louis, MO, USA) and cloxacillin (CLX) (Polfa Tarchomin, Warsaw, Poland) was added, along with a bacterial inoculum with a density of 0.5 on the McFarland scale. The concentrations of the carboxylic acids and CLX were experimentally determined [30] and are presented in Supplementary Materials—Table S1. They range from 1/16 MIC (256 mg/L) to 1/256 MIC (16 mg/L). The prepared culture medium was then incubated at 37 ± 1 °C for a period of 16 ± 2 h under aerobic conditions.

After incubation, the entire culture was centrifuged (6000 RCF) for 5 min. The supernatant was then removed, and the pellet was resuspended in 800 µL of Fenozol (A&A Biotechnology, Gdynia, Poland) and subjected to homogenization (Bertin Technologies SAS, Minilys, Montigny-le-Bretonneux, France). The homogenization process (2800 RCF, 30 s) was repeated three times.

### 2.3. Reverse Transcription

Reverse transcription was performed using the Omniscript Reverse Transcription Kit (Qiagen, Hilden, Germany) according to the manufacturer’s protocol. To the reaction well, 10× RT buffer, dNTP mix, Oligo-dT primer (EURx Ltd., Gdańsk, Poland), Ribonuclease Inhibitor (EURx Ltd., Gdańsk, Poland), and Omniscript Reverse Transcriptase were added. Next, the template RNA was introduced. The RT reaction was carried out at 37 °C for 60 min [32].

### 2.4. Real-Time PCR Reaction

To achieve the objective of the study, which was to determine the expression of the genes *icaA*, *icaB*, *icaC*, *icaR*, and *gyrB*, Real-Time PCR was employed [32]. The experiments were conducted using the CFX96 Touch Real-Time PCR Detection System (Bio-Rad, Feldkirchen, Germany). In the reaction well, 5 µL of SsoAdvanced Universal SYBR Green Supermix (Bio-Rad, Germany), 3 µL of diluted cDNA template, 1.4 µL of water, and 0.3 µL of each primer were added—Table S2. The total volume was 10 µL. The sequences of the primers used are provided in the Supplementary Materials. Each measurement was carried out in triplicate.

The reaction was performed in the following setup:

1. Pre-incubation 98 °C 30 s;
2. Amplification with denaturation 95 °C for 10 s (40 cycles);
3. Annealing at 60 °C for 30 s;
4. Final melting from 65 °C to 95 °C—increase of 0.5 °C every 5 s.

### 2.5. Determination of Bacterial Viability

In this section, carboxylic acids were used at concentrations ranging from 1/16 MIC (256 mg/L) to 1/256 MIC (16 mg/L). Additionally, a positive control was also used—a sample not treated with antibiotics and organic acids. The culture media and the course of cultivation were prepared exactly as described in Section 2.2. After incubation at a temperature of 37 ± 1 °C for a period of 16 ± 2 h under aerobic conditions, the culture medium was removed, and viability was determined using the Microbial Viability Assay Kit-WST according to the manufacturer’s instructions (Dojindo Laboratories, Kumamoto, Japan) [33]. The analysis was performed using a Multiskan EX Microplate Reader (Thermo Electron Corp., Vantaa, Finland).

### 2.6. Determination of the Anti-Biofilm Activity of Selected Compounds and Their Combinations

To 190 µL of TSB medium supplemented with 0.5% glucose, 10 µL of bacterial suspension of the tested strains was added at a concentration of 1–2 × 10^8^ CFU/mL, corresponding to a turbidity of 0.5 on the McFarland scale. Two clinical *Staphylococcus epidermidis* strains were selected for the study, both showing high phenotypic ability to form biofilm (strong biofilm strains) and different susceptibility phenotypes (MSSE and MRSE). The phenotypic biofilm-forming ability of each strain was assessed by measuring optical density (λ = 570 nm) using an automated spectrophotometer, Multiskan EX Microplate Reader (Thermo Electron Corp., Vantaa, Finland). Each isolate was classified as a strong biofilm producer (OD_570_ ≥ 1), a moderate/intermediate biofilm producer (0.1 ≤ OD_570_ < 1), or a non-biofilm producer (OD_570_ < 0.1) based on the optical density measurements [34]. After incubation (at 37 ± 1 °C for 16 ± 2 h under aerobic conditions), the native culture medium was removed and replaced with TSB (with 0.5% glucose), supplemented with selected chemical compounds or their combinations. Among the organic acids, CA, p-CA and FA were selected (Aldrich Chemistry, Saint Louis, MO, USA), as well as CLX (Polfa Tarchomin, Warsaw, Poland). The combinations consisted of mixtures of CLX (at a concentration of 0.6 mg/L) and CA, p-CA and FA (at concentrations of 0.3, 0.6, and 1.2 mg/L)—Figure S1. The sub-MIC concentrations used corresponded to the substance ratios used in previous determinations and to the EUCAST recommendations. The thus enriched medium was subjected to another incubation (at 37 ± 1 °C for 16 ± 2 h under aerobic conditions). After incubation, the mass of the formed biofilm was evaluated using a modified Christensen method [35], and cell viability was assessed using the Microbial Viability Assay Kit-WST according to the manufacturer’s instructions (Dojindo Laboratories, Kumamoto, Japan). The organization of the examined plate is presented in the Supplementary Materials.

### 2.7. Statistical Analysis

All experiments were performed in triplicate. Data are presented as mean ± standard deviation. Due to the exploratory nature of the study and the observed variability of gene expression under sub-MIC conditions, statistical analysis was applied descriptively to identify trends rather than to draw definitive quantitative conclusions [33].

### 2.8. Scanning Laser Confocal Microscopy

The assay was performed in a sterile 50 mm Petri dish with a sterile coverslip placed at the center, following aseptic procedures [13]. To the prepared incubation chamber, 1.45 mL of antibiotic solution and 1.45 mL of the selected carboxylic acid (CA, p-CA, FA or SA) solution at the appropriate concentrations (FIC or ½ FIC) were added. FIC values were calculated using the following formulas: CLX FIC = MIC of CLX with CA, p-CA, FA or SA/MIC of CLX; FIC of CA, p-CA, FA or SA = MIC of CA, p-CA, FA or SA with CLX/MIC of CA, p-CA, FA or SA. The next step involved the inoculation of 0.1 mL of the bacterial suspension of the test strain in a 0.9% NaCl solution, corresponding to 0.5 McFarland (1–2 × 10^8^ CFU/mL). The culture was incubated at 37 ± 1 °C for 18 h. After incubation, the culture was stained with Syto™ 9 Green, a fluorescent nucleic acid stain (Invitrogen, Eugene, OR, USA), and propidium iodide (MERCK/Sigma-Aldrich, Darmstadt, Germany), according to the manufacturer’s instructions, and analyzed using the Nikon Eclipse Ti-E A1R-SI device (Nikon Instruments, Amsterdam, The Netherlands). The assay was performed in duplicate. The concentrations used in the antibiotic-acid combinations are provided in Table 1.

## 3. Results

### 3.1. Gene Expression Analysis

The gene expression analysis was performed using a single *S. epidermidis* strain, and the experiments were conducted exclusively under in vitro conditions. Given the known strain-dependent variability in biofilm formation and gene regulation, the findings should be regarded as strain-specific and exploratory.

**Figure 2:**
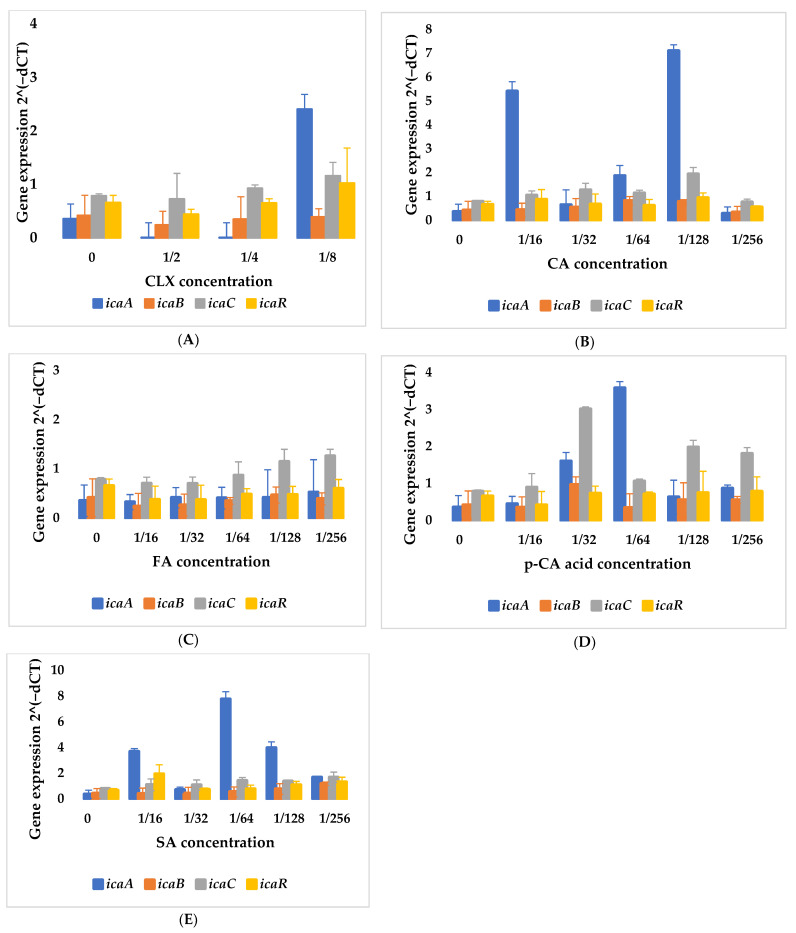
Analysis of *icaA*, *icaB*, *icaC*, and *icaR* gene expression in *S. epidermidis* bacteria, depending on the concentration of cloxacillin (**A**), cinnamic acid (**B**), ferulic acid (**C**), *p*-coumaric acid (**D**), sinapic acid (**E**), used at decreasing concentration levels (fractions of MIC); CLX—cloxacillin; CA—cinnamic acid; FA—ferulic acid; p-CA—*p*-coumaric acid; SA—sinapic acid; dCT—CT of the tested gene − CT of the reference gene.

Gene expression profiles of *icaA*, *icaB*, *icaC*, and *icaR* in response to CLX and CA, p-CA, FA and SA are presented in Figure 2. The point labeled “0” corresponds to the untreated control [20]. Overall, the profiles revealed variable, non-uniform responses across sub-MIC concentrations rather than consistent dose-dependent trends.

A particularly noteworthy finding is that the highest expression levels in individual samples were exhibited by different genes (most frequently *icaA*), whereas the lowest expression was observed, in most cases, for the *icaB* gene, which is responsible for deacetylation. Decreasing concentrations of CLX (Figure 2A) promoted an increase in the expression of all genes. The increase in *icaA* expression at a concentration of 1/8 MIC is particularly pronounced.

In the case of CA (Figure 2B), the greatest variability in expression levels was observed for the *icaA* gene. This gene is a key component of the *ica* operon, responsible for the synthesis of poly-N-acetylglucosamine (PIA). The expression of the remaining genes was at a similar level, regardless of the CA concentration used.

The expression profile of genes in the presence of FA was characterized by a consistently low expression of *icaA* and *icaB* (Figure 2C). No increase in *icaA* gene expression was observed in this case.

Distinct expression patterns were observed in the presence of *p*-CA (Figure 2D). At a concentration of 1/32 MIC, the highest increases in *icaB* and *icaC* gene expression were observed, while the expression levels of *icaA* and *icaR* were lower. Upon a twofold dilution (1/64 MIC), a decrease in the expression of *icaB* and *icaC* genes was noted, accompanied by the highest increase in *icaA* expression. The expression of *icaC* at concentrations of 1/128 MIC and 1/256 MIC increased again.

Relatively similar results to CA were obtained for SA (Figure 2E), with the most substantial increase in *icaA* expression occurring at 1/16 MIC and 1/64 MIC. The expression of the *icaA* gene at a concentration of 1/64 MIC is the highest among all genes tested for all substances.

It should be noted that the observed changes in the expression of the analyzed genes did not show a consistent concentration-dependent pattern—the response was nonlinear and strain-specific, which highlights the complexity of the system and the need for further studies. The expression of the *icaR* gene in all four tested substances (CA, FA, p-CA and SA) was at a relatively similar level.

#### 3.1.1. Determination of Viability

**Figure 3:**
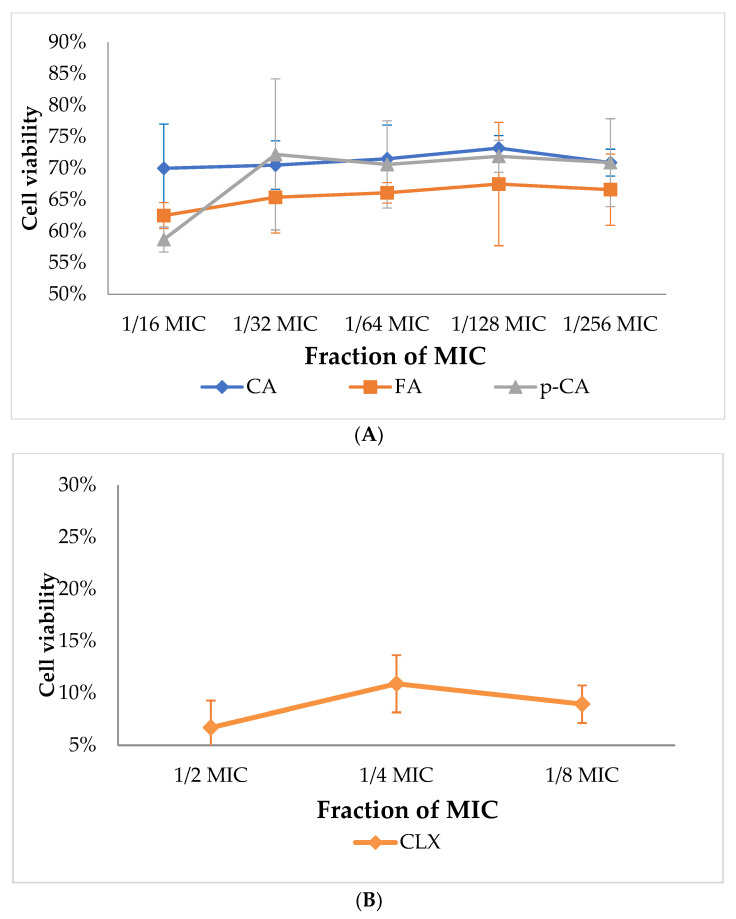
Determination of viability of the tested *S. epidermidis* strain for cinnamic acid, ferulic acid and *p*-coumaric acid within the concentration range from 1/16 MIC to 1/256 MIC (**A**) and for cloxacillin at concentrations ranging from 1/2 MIC to 1/8 MIC (**B**); CLX—cloxacillin; CA—cinnamic acid; FA—ferulic acid; p-CA—*p*-coumaric acid.

The next stage of the study was the determination of the viability of the tested *S. epidermidis* strain in an environment supplemented with selected compounds. This step was conducted for comparison purposes at the same concentration ranges as in the molecular section. SA was not included in this section due to its least pronounced antibacterial effect among all the substances tested. Viability was assessed under sub-MIC conditions to determine whether the observed transcriptional responses occurred independently of strong antibacterial effects. The concentrations of the substances used in the experiment were the same as those applied in the molecular aspect of the conducted study—ranging from 1/16 MIC to 1/256 MIC for CA, FA and p-CA and from 1/2 MIC to 1/8 MIC for CLX. The obtained results are presented as percentage values in comparison with the growth control. The viability determinations for CA, FA and p-CA are presented in Figure 3A, while for CLX in Figure 3B.

The viability assessment using the WST test indicated a weak inhibitory effect on the growth of the tested bacterial strains (55–75% of cell viability) at concentrations of CA, FA, p-CA below their MIC values (1/16 MIC, 1/32 MIC, 1/64 MIC, 1/128 MIC and 1/256 MIC). High antimicrobial activity of CLX (5–15% of cell viability) was confirmed, even at concentrations corresponding to 1/8 MIC. The most linear, inversely proportional relationship between concentration and viability was demonstrated by FA. A low, linear relationship was shown by p-CA and CLX. All obtained determinations appear to indicate the lack of a simple, linear relationship between the concentration of the tested substances and cell viability.

#### 3.1.2. Determination of Preformed Biofilm Eradication Activity of Cinnamic Acid (CA), *p*-Coumaric Acid (p-CA) and Ferulic Acid (FA) in Combination with Cloxacillin (CLX)

**Figure 4:**
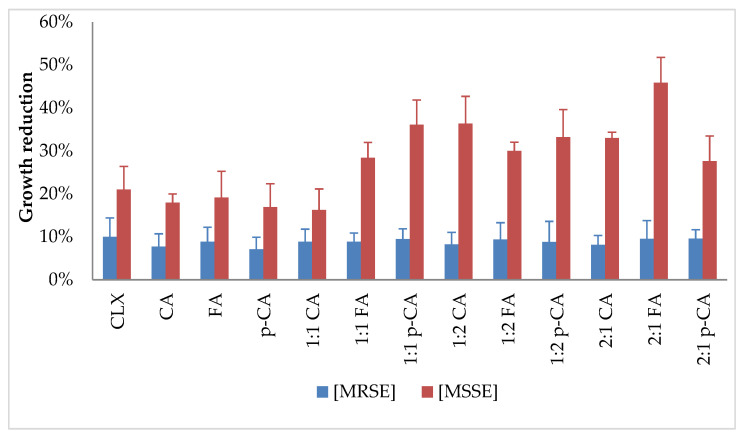
Biofilm mass reduction after treatment with tested substances at selected concentrations; determination of concentrations: 1:1 cloxacillin and acid—both 0.6 mg/L; 1:2 cloxacillin—0.6 mg/L, acid—1.2 mg/L; 2:1 cloxacillin—0.6 mg/L, acid—0.3 mg/L; CLX—cloxacillin; CA—cinnamic acid; FA—ferulic acid; p-CA—*p*-coumaric acid.

**Figure 5:**
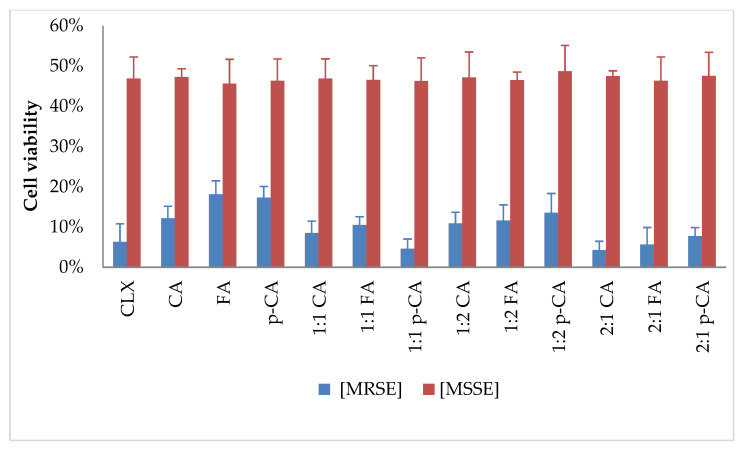
Reduction of cell viability after treatment with tested substances at selected concentrations; determination of concentrations: 1:1 cloxacillin and acid—both 0.6 mg/L; 1:2 cloxacillin—0.6 mg/L, acid—1.2 mg/L; 2:1 cloxacillin—0.6 mg/L, acid—0.3 mg/L; CLX—cloxacillin; CA—cinnamic acid; FA—ferulic acid; p-CA—*p*-coumaric acid.

In the next stage, unlike in the studies in the previous publication [13], the effect of the tested compounds on preformed biofilm was examined in vitro. Solutions with the specified concentrations were used to supplement bacterial cultures with preformed biofilms. After further incubation, the reduction in the formed biofilm and cell viability was determined in relation to growth control. The obtained results are presented in the figures below (Figure 4 and Figure 5).

The results obtained varied significantly depending on the antibiotic susceptibility profile of the strain tested. For the MRSE strain, the biofilm mass reduction ranged from 7.12% to 9.9%, and cell viability ranged from 4.29% to 18.11%. For the MSSE strain, the use of single substances resulted in a biofilm mass reduction ranging from 16.94% to 21%, while the use of combinations resulted in a reduction ranging from 27.64% to 45.85%. The results for viability reduction were much more uniform, ranging from 45.60% to 48.70% for all combinations. It is worth noting that the reduction in viability and biofilm mass was comparable or even higher for the combinations than for each substance alone, and the antibiotic concentrations used were significantly lower than those resulting from the MIC determination.

### 3.2. Confocal Microscopy

**Figure 6:**
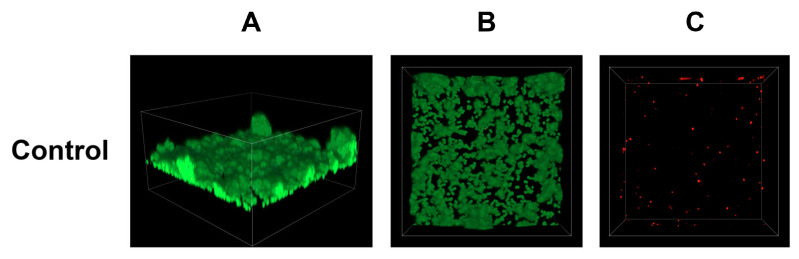
Representative confocal microphotographs of control (untreated) *S. epidermidis* biofilm. Z-stack 3D reconstruction (**A**), top view in SYTO 9—green channel (**B**), top view in propidium iodide—red channel (**C**).

**Figure 7:**
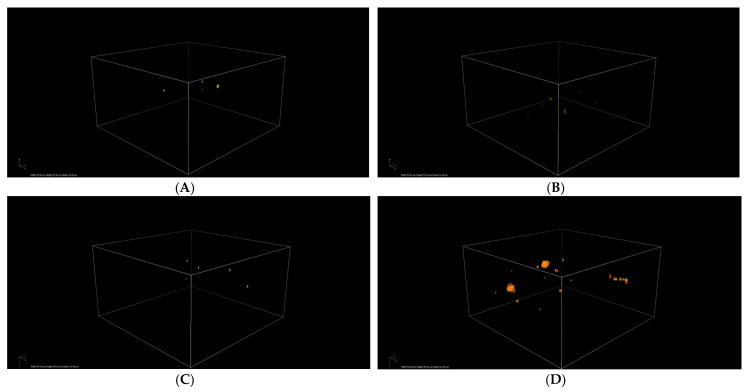
Confocal images (Z-stack 3D reconstruction in SYTO 9—green and propidium iodide—red channel) of *S. epidermidis* biofilm exposed to a combination of cloxacillin and: (**A**)—*p*-coumaric acid; (**B**)—ferulic acid; (**C**)—cinnamic acid; (**D**)—sinapic acid in FICs; CLX—cloxacillin; CA—cinnamic acid; FA—ferulic acid; p-CA—*p*-coumaric acid; SA—sinapic acid.

**Figure 8:**
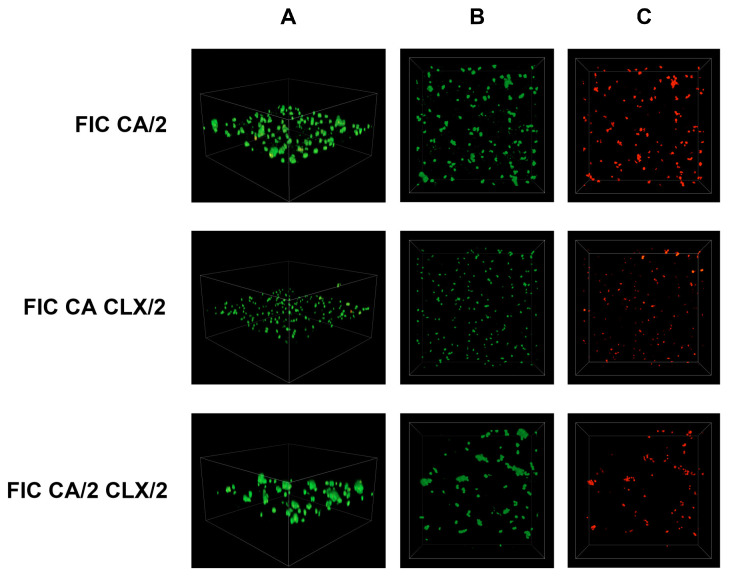
Representative confocal microphotographs of *Staphylococcus epidermidis* biofilm exposed to a combination of cloxacillin and cinnamic acid at various dilutions: FIC CA/2—1/2 FIC of cinnamic acid and FIC of cloxacillin; FIC CA CLX/2—FIC of cinnamic acid and 1/2 FIC of cloxacillin; FIC CA/2 CLX/2—both substances in 1/2 FIC. Z-stack 3D reconstruction (**A**), top view in SYTO 9—green channel (**B**), top view in propidium iodide—red channel (**C**); CLX—cloxacillin; CA—cinnamic acid.

**Figure 9:**
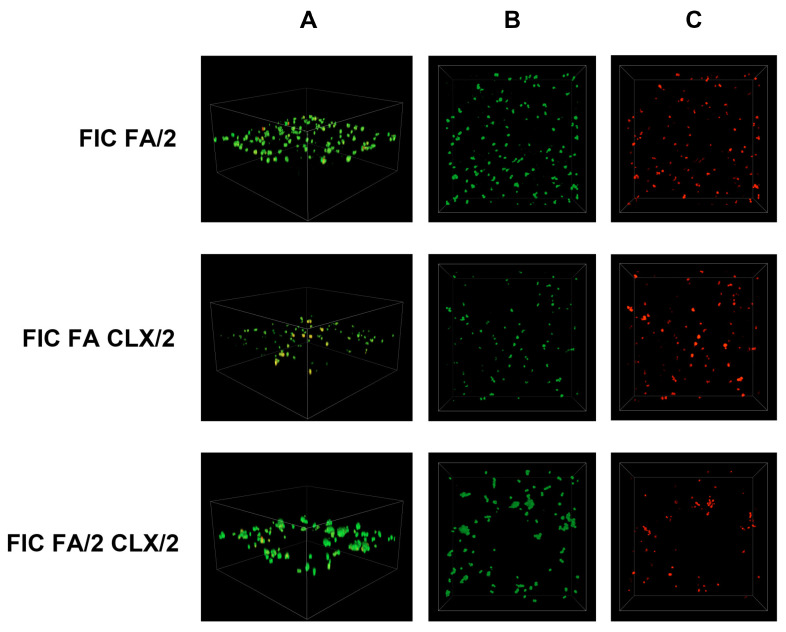
Confocal images of *S. epidermidis* biofilm exposed to a combination of cloxacillin and ferulic acid at various dilutions: FIC FA/2—1/2 FIC of ferulic acid and FIC of cloxacillin; FIC FA CLX/2—FIC of ferulic acid and 1/2 FIC of cloxacillin; FIC FA/2 CLX/2—both substances in 1/2 FIC. Z-stack 3D reconstruction (**A**), top view in SYTO 9—green channel (**B**), top view in propidium iodide—red channel (**C**); CLX—cloxacillin; FA—ferulic acid.

**Figure 10:**
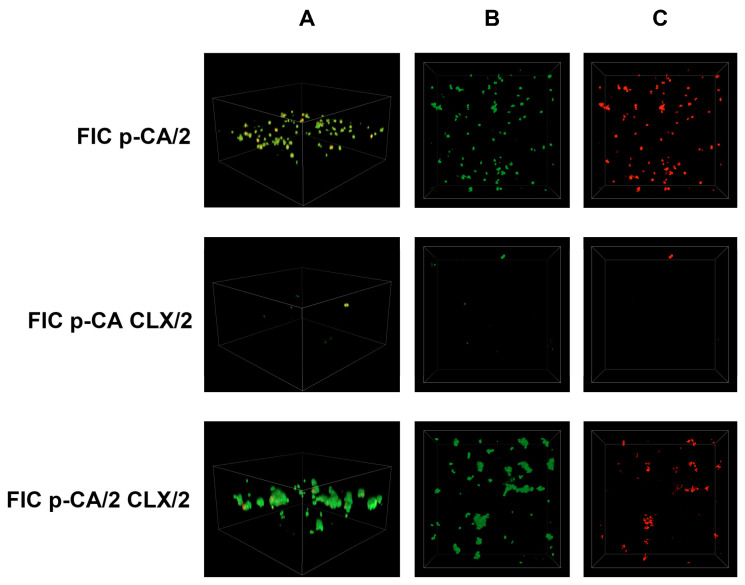
Confocal images of *S. epidermidis* biofilm exposed to a combination of cloxacillin and *p*-coumaric acid at various dilutions: FIC p-CA/2—1/2 FIC of *p*-coumaric acid and FIC of cloxacillin; FIC p-CA CLX/2—FIC of *p*-coumaric acid and 1/2 FIC of cloxacillin; FIC p-CA/2 CLX/2—both substances in 1/2 FIC. Z-stack 3D reconstruction (**A**), top view in SYTO 9—green channel (**B**), top view in propidium iodide—red channel (**C**); CLX—cloxacillin; p-CA—*p*-coumaric acid.

**Figure 11:**
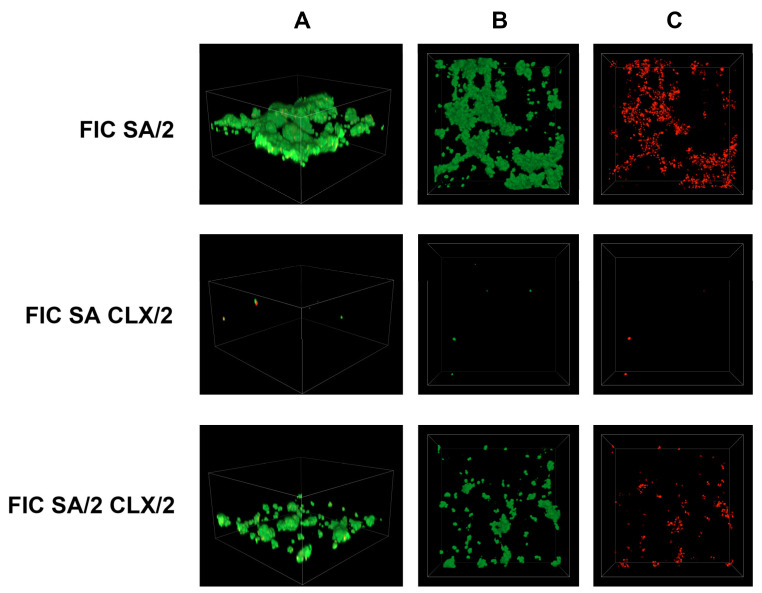
Confocal images of *S. epidermidis* biofilm exposed to a combination of cloxacillin and sinapic acid at various dilutions: FIC SA/2—1/2 FIC of sinapic acid and FIC of cloxacillin; FIC SA CLX/2—FIC of sinapic acid and 1/2 FIC of cloxacillin; FIC SA/2 CLX/2—both substances in 1/2 FIC. Z-stack 3D reconstruction (**A**), top view in SYTO 9—green channel (**B**), top view in propidium iodide—red channel (**C**); CLX—cloxacillin; SA—sinapic acid.

Simultaneously, an analysis of *S. epidermidis* biofilm formation was conducted using confocal laser scanning microscopy. In this study, previously determined FIC (fractional inhibitory concentration) values were applied, and the following combinations were tested: antibiotic (FIC)—acid (FIC), antibiotic (FIC)—acid (1/2 FIC), antibiotic (1/2 FIC)—acid (FIC), and antibiotic (1/2 FIC)—acid (1/2 FIC). The control sample consisted of *S. epidermidis* cultured in TSB liquid medium supplemented with 0.5% glucose. The obtained results are presented in Figure 6, Figure 7, Figure 8, Figure 9, Figure 10 and Figure 11.

In the growth control (Figure 6), the biofilm mass covered almost the entire tested surface, and no signals were visible in the dead cell channel. The 3D reconstruction also reflects the three-dimensional structure of the biofilm mass produced during incubation. This measurement was performed for comparison with surfaces treated with various combinations of cinnamic acid (CA) and its derivatives (FA, p-CA, and SA) with cloxacillin (CLX).

Figure 7 presents three-dimensional images of the biofilm mass. Only a few cells stained with propidium iodide are visible, indicating that they are dead. Individual images clearly demonstrate a reduction in bacterial cell counts by the combinations of the tested substances (CA, FA, p-CA, SA) with CLX at MIC concentrations.

The use of cinnamic acid (CA) resulted in a reduction in the mass of the biofilm exposed to this substance in combination with cloxacillin (CLX) at various sub-FIC combinations (Figure 8). The number of dead cells detected by the propidium iodide channel also significantly increased compared to the growth control.

The figures also show a visible dispersion of live cells stained with SYTO 9 when CA is used in FIC and CLX at FIC/2 concentration. In comparison, the two other combinations show live cells clustered together in larger “clusters.” This may be due to the greater ability of this combination of substances to eradicate biofilm—bacterial cell clusters.

Very similar results were obtained for the combination of ferulic acid (FA) with cloxacillin (CLX). The FIC FA and 1/2 FIC CLX concentrations resulted in greater dispersion of viable bacterial cells than in the other combinations of these two substances (Figure 9). However, in each of the combinations in Figure 9, a clear difference in the amount is visible compared to the growth control.

Figure 10 shows the combination of p-CA with CLX at sub-FICs. The FIC of p-CA and 1/2 FIC of CLX resulted in the highest reduction in bacterial cell count and biofilm mass among all the acids tested (Figure 10; *FIC p-CA CLX/2*). In this case, the figure shows single-cell clusters. In the other combination options, the cell counts are comparable to those shown in the previous figures.

A similar situation can be observed for the combination of FIC SA and 1/2 FIC CLX in Figure 11. This combination shows single small-cell clusters. However, the other two options in Figure 11 show a higher bacterial cell count and biofilm mass compared to the other substances used (Figure 8, Figure 9 and Figure 10). The number of viable bacterial cells is also significantly higher in this case.

## 4. Discussion

Several studies have investigated the antimicrobial activity of CA and its derivatives, but information on their effects on *S. epidermidis*, both in planktonic and biofilm cultures, is very limited. Similar studies to those mentioned in this publication were conducted by the team of Mastoor et al. [36]. They also investigated the effect of cinnamic acid and its derivatives on the expression of, among others, the *icaA* gene. During their studies, they observed non-linear changes in *icaA* gene expression due to the use of cinnamic acid and its derivatives. It is worth noting, however, that these studies were conducted at concentrations equal to the MIC. In our study, sub-MIC concentrations were used. The use of a different species (*S. aureus*) and the absence of glucose in the medium certainly contributed to the differences observed.

The influence of another narrow-spectrum semisynthetic penicillin was investigated by Sharafi et al., who studied the effect of oxacillin (a derivative of which is CLX) on bacterial growth, biofilm production, and gene expression. They observed an increase in biofilm mass and a decrease in *icaA* gene expression with decreasing antibiotic concentrations, and combining the substance with antibiotics at a lower concentration gives a better effect than using the substance separately [32]. Similar studies were conducted by Mirzaei et al., who analyzed the impact of CLX on gene expression, including *icaA*, and reported an increase in its expression at sub-MIC concentrations. In this case, even the use of the same test species and the addition of glucose to the medium allowed for obtaining a linear relationship [37]. Conversely, Minich et al. observed a decrease in *icaA* mRNA expression at sub-MIC (1/2 MIC) concentrations of oxacillin. The use of oxacillin alone at 1/2 MIC resulted in a smaller decrease in expression than administration of vanillin alone at a subinhibitory concentration. However, combining the two substances at subinhibitory concentrations also resulted in a significant decrease in expression. This may indicate a greater contribution of vanillin to the inhibition of *icaA* gene expression compared to oxacillin [38].

In our study, a low expression level of *icaA* was observed up to a concentration of 1/4 MIC. Only further reduction in the antibiotic concentration (below 1/4 MIC) was associated with an increase in *icaA* expression, which coincided with enhanced biofilm formation observed under the same conditions. The observed effect suggests an influence of cloxacillin on gene expression, which is consistent with the observations of other authors studying this phenomenon.

The expression of the *icaA* gene under the influence of various CA derivatives was studied by Nuryastuti et al. They reported a significant increase in *icaA* expression in the presence of sub-MIC concentrations of cinnamon oil [39].

Interestingly, gene expression differed significantly for structurally related acids—*p*-CA and FA—with expression levels nearly twice as low, particularly for *icaA* and *icaC*. Divergent results were presented by Kot et al., who analyzed *S. aureus* strains and observed a substantial decrease in *icaA* and *icaD* expression in cultures containing 1/2 MIC CA in strains with high adherence capacity, whereas strains with low biofilm-forming capacity exhibited a twofold decrease in gene expression. In cultures containing 1/4 MIC trans-cinnamaldehyde, a decrease in *icaD* expression was also observed for both high- and low-adherence strains. No changes in *icaA* expression were noted for any strains cultured with 1/4 MIC trans-cinnamaldehyde [40]. This work also indicates the importance of the timing of expression measurement from the start of the study. There were differences in expression levels at 3 and 12 h of the experiment under the same conditions.

Chen et al. demonstrated that an ethanolic extract of *Sanguisorba officinalis* L. (*S. officinalis*) inhibited the expression of the *icaADBC* operon genes, except for the regulatory *icaR* gene, whose expression was upregulated [41]. It should be noted that CA and its derivatives are widely distributed among plant species and are present in *S. officinalis* extracts. The findings by Chen et al. confirm the influence of selected CA derivatives on gene expression. In our study, no reduction in *icaADBC* gene activity was observed alongside an increase in *icaR* activity. This discrepancy may be due to differences in the concentrations of the substances tested, different interactions between them, or alternative mechanisms influencing gene expression. Future studies should investigate the role of the *icaR* regulatory network in mediating these strain-specific responses to CA derivatives.

Gene expression in *S. epidermidis* is strongly phase-dependent and can fluctuate independently of the compounds tested. As a result, the described “nonlinear” expression profiles may simply reflect physiological variability during the growth cycle rather than compound-specific regulation. The presented data provide preliminary insight into the transcriptional responses of *S. epidermidis* to sub-MIC levels of CA derivatives rather than definitive mechanistic evidence.

The verification of viability in the present work was performed using CLSM methods and indicated a linear, inversely proportional relationship between the concentration of the substances and the number of live cells at concentrations corresponding to a fraction of the MIC value [13,30]. In contrast, the study by Firmino et al. showed that, for comparison, the viability of cinnamic aldehyde at concentrations of 1/16 MIC and lower was >80%. These studies were conducted on *S. aureus* and *S. epidermidis* strains [42]. In this study, after the application of CA, FA and p-CA against the *S. epidermidis* strain, the viability was in the range of approximately 60–70% (Figure 3A). In this case, the species of bacteria used may have an impact on this subtle difference.

Infections associated with the colonization of implants used in medicine are a serious problem of modern medicine and a great therapeutic challenge. The biofilm formed on their surface causes inflammation that hinders the rehabilitation process. Moreover, during its growth, the biofilm structure becomes unstable and may disintegrate, leading to bacteremia that poses a threat to the patient’s health and life. At present, eradication of an established biofilm is impossible, and the only therapeutic option is the removal of the colonized implant.

This clinical challenge motivated an exploratory assessment of the effects of the tested compounds on preformed biofilms under in vitro conditions. The study evaluated the effects of cloxacillin and cinnamic acid (CA), *p*-coumaric acid (p-CA) and ferulic acid (FA) at concentrations corresponding to the MIC, as well as in combination with cloxacillin at a concentration of 0.6 mg/L. For comparison, Tian et al. reported a biofilm removal rate of approximately 60% for the combination of CA and berberine. In this study, the inhibition of biofilm formation by CA was approximately 20%, while in the combination of CA and CLX (2:1), the biofilm mass reduction was approximately 35% [43]. Mastoor et al., while testing the anti-biofilm activity of CA and its derivatives against *S. aureus* strains, obtained inhibition of biofilm formation by these substances at the level of 15–100%. The best results were determined for trans-4-nitrocinnamic acid, where biofilm reduction was at the level of 71–88%, depending on the strain tested [36]. In the study by Borges et al., FA had a preventive effect on biofilm formation and promoted a reduction in biofilm activity by >70% [44]. As can be seen in the above-mentioned studies, the inhibition of the biofilm formation process occurs at different levels, depending on the substances used, the incubation time and the medium used.

The confocal microscopy observations suggest qualitative alterations in biofilm architecture and a reduction in metabolically active cells following exposure to selected combinations of CLX and CA derivatives. Differences between FIC and sub-FIC combinations were apparent at the structural level; however, these observations are based exclusively on qualitative visualization. An interesting phenomenon is also the considerable difference between growth at concentrations corresponding to the FIC and sub-FICs in various combinations. Notably, combinations in which the acid concentration exceeded that of the antibiotic appeared to be associated with more pronounced qualitative biofilm disruption than the inverse ratios. The combination of p-CA at a concentration of 512 mg/L (FIC) with CLX at a concentration of 4 mg/L (1/2 FIC) demonstrated more pronounced qualitative biofilm disruption than the combination of p-CA at 256 mg/L (1/2 FIC) and CLX at 8 mg/L (FIC). This effect is particularly intriguing when considering the fact that the antibacterial activity of CLX is significantly higher than that of any of the acids studied. The same qualitative pattern was observed in an independent experiment. Albano et al. confirmed in their study that sub-MIC concentrations of cinnamic aldehyde weakened biofilm formation by *S. epidermidis* strains and destroyed the already formed structure. Images obtained using confocal laser scanning microscopy (CLSM) illustrated the effect of cinnamic aldehyde on the separation and destruction of existing biofilms [45].

In this study, confocal microscopy was applied solely for qualitative visualization of biofilm architecture. Owing to methodological constraints, additional imaging and quantitative analyses could not be performed. Consequently, this component is presented as a proof-of-concept. Subsequent stages should involve quantitative studies, allowing for a precise assessment of the effects of the tested substances on the metabolic activity and structure of the biofilm.

All phenolic acids tested in this study (CA, p-CA, FA and SA), acting alone but also in combination with antibiotics, show antibacterial and anti-biofilm activity [46,47]. The results obtained in this study do not allow for a definitive conclusion regarding the specific effects of the analyzed compounds (both the antibiotic and the tested carboxylic acids) on the expression of the *icaADBC* operon genes and, consequently, on biofilm formation processes. Further research appears justified to elucidate the influence of these compounds on the molecular aspects of biofilm formation, including their effect on the expression of biofilm-associated genes independent of the *icaADBC* operon, using a larger number of *S. epidermidis* strains.

## 5. Conclusions

In conclusion, the present study provides exploratory insight into the effects of sub-MIC concentrations of cloxacillin (CLX) and selected cinnamic acid (CA) derivatives (p-CA, FA, SA) on biofilm formation, gene expression, and biofilm architecture in a clinical methicillin-resistant *S. epidermidis* (MRSE) strain. The observed responses were complex and non-linear, did not consistently follow concentration-dependent trends, and differed among structurally related compounds.

While the observed effects indicate potential interactions between β-lactam antibiotics and cinnamic acid derivatives, further studies involving multiple strains, quantitative biofilm analyses, and mechanistic investigations are required before any conclusions regarding therapeutic applicability can be drawn.

The subsequent stages of the presented experiment will involve quantitative measurement of biofilm formation following the use of cinnamic acid and its derivatives in combination with cloxacillin, using confocal microscopy. It will be reasonable to include further clinical strains in order to perform statistical calculations. It is also planned to examine the expression of genes related to biofilm production independent of the *icaADBC* operon.

## Figures and Tables

**Table 1:** Calculated FIC of cinnamic acid and its derivatives with cloxacillin used.

FIC Antibiotic—FIC Acid [mg/L]				FIC Antibiotic—1/2 FIC Acid [mg/L]			
CLX	8	CA	256	CLX	8	CA	128
	8	FA	256		8	FA	128
	8	p-CA	512		8	p-CA	256
	8	SA	512		8	SA	256
**1/2 FIC Antibiotic** **—FIC acid [mg/L]**				**1/2 FIC Antibiotic** **—1/2 FIC acid [mg/L]**			
CLX	4	CA	256	CLX	4	CA	128
	4	FA	256		4	FA	128
	4	p-CA	512		4	p-CA	256
	4	SA	512		4	SA	256

CLX—cloxacillin; CA—cinnamic acid; FA—ferulic acid; p-CA—*p*-coumaric acid; SA—sinapic acid.

## Data Availability

The original contributions presented in this study are included in the article/Supplementary Materials. Further inquiries can be directed to the corresponding author.

## Supplementary Materials

The following supporting information can be downloaded at: https://www.mdpi.com/article/10.3390/cimb48030336/s1.

## Abbreviations

CoNS	coagulase-negative staphylococci
MRSE	methicillin-resistant *Staphylococcus epidermidis*
MSSE	methicillin-susceptible *Staphylococcus epidermidis*
CA	cinnamic acid
p-CA	*p*-coumaric acid
SA	sinnapic acid
FA	ferulic acid
CLX	cloxacillin
FIC	Fractional Inhibitory Concentration
MIC	Minimal Inhibitory Concentration
PIA	polysaccharide intercellular adhesin
PNAG	poly-β-N-acetylglucosamine
sub-MIC	sub-Minimal Inhibitory Concentration
