# Supplementary material for: Anti-Biofilm Activity of Combinations of Cinnamic Acid and Its Derivatives with Cloxacillin Against Methicillin-Resistant Staphylococcus epidermidis
Source: Curr Issues Mol Biol. 2026 Mar 23;48(3):336. doi: 10.3390/cimb48030336 (PMC13025280; doi:10.3390/cimb48030336)
Supplement: Supplementary file 1 [file cimb-48-00336-s001.zip › Figure S1.pdf]

CLX b	CA b	FA b	p-CA b	1:1 CA b	1:1 FA b	1:1 p-CA b	1:2 CA b	1:2 FA b	1:2 p-CA b	GC	
CLX	CA	FA	p-CA	1:1 CA	1:1 FA	1:1 p-CA	1:2 CA	1:2 FA	1:2 p-CA	GC	
CLX	CA	FA	p-CA	1:1 CA	1:1 FA	1:1 p-CA	1:2 CA	1:2 FA	1:2 p-CA	GC	
CLX	CA	FA	p-CA	1:1 CA	1:1 FA	1:1 p-CA	1:2 CA	1:2 FA	1:2 p-CA	GC	
2:1 CA	2:1 FA	2:1 p-CA	NC	NC	NC	NC	NC	NC	NC	GC	
2:1 CA	2:1 FA	2:1 p-CA	NC	NC	NC	NC	NC	NC	NC	GC	
2:1 CA	2:1 FA	2:1 p-CA	NC	NC	NC	NC	NC	NC	NC	GC	

Figure S1. Organisation of plate: 1:1 cloxacillin and acid—both 0.6 mg/L; 1:2 cloxacillin—0.6 mg/L, acid—1.2 mg/L; 2:1 cloxacillin—0.6 mg/L, acid—0.3 mg/L; CLX—cloxacillin; CA—cinnamic acid; FA—ferulic acid; p-CA—*p*-coumaric acid; GC—growth control; NC—sterility control; the letter b denotes the supplemented medium without the addition of bacteria.
