# Supplementary material for: Anti-Biofilm Activity of Combinations of Cinnamic Acid and Its Derivatives with Cloxacillin Against Methicillin-Resistant Staphylococcus epidermidis
Source: Curr Issues Mol Biol. 2026 Mar 23;48(3):336. doi: 10.3390/cimb48030336 (PMC13025280; doi:10.3390/cimb48030336)
Supplement: Supplementary file 1 [file cimb-48-00336-s001.zip › Table S1.pdf]

Table S1. Concentrations of cinnamic acid and its derivatives and cloxacillin used for bacterial RNA isolation.

**Substance**	**Concentration [mg/L]**				
**cinnamic acid**	256	128	64	32	16
**ferulic acid**	256	128	64	32	16
**p-coumaric acid**	256	128	64	32	16
**sinapic acid**	256	128	64	32	16
**cloxacillin**	256	128	64	X	X
