# Supplementary material for: Anti-Biofilm Activity of Combinations of Cinnamic Acid and Its Derivatives with Cloxacillin Against Methicillin-Resistant Staphylococcus epidermidis
Source: Curr Issues Mol Biol. 2026 Mar 23;48(3):336. doi: 10.3390/cimb48030336 (PMC13025280; doi:10.3390/cimb48030336)
Supplement: Supplementary file 1 [file cimb-48-00336-s001.zip › Table S2.pdf]

Table S2. Sequences of primers used in the RT-PCR reaction.

Gene	Oligonucleotide Sequence	Amplimer Length [bp]	Ref.
icaA	Forward: 5′TGGTTGTATCAAGCGAAGTCA3′<br>Reverse: 5′ATCCTCAGTAATCATGTCAGTATCC3′	127	[3]
icaB	Forward: 5′CTGTCACACCAGATGCCGATAACTA3′<br>Reverse: 5′CCGTCCCATTCCTTTATTAGCGTTTC3′	88	[3]
icaC	Forward: 5′GGCGTCGGAATGATGTTAAGAGA3′<br>Reverse: 5′AGTTAGGCTGGTATTGGTCAAATTGT3′	94	[3]
icaR	Forward: 5′GCGATGTGCGTAGGATCATAA3′<br>Reverse: 5′TGTTCAATTATCTAGTGCTCCAGAAG3′	117	[3]
gyrB	Forward: 5′TGGTGCTGGACAGATACAAGT3′<br>Reverse: 5′CCTGCTAATGCCTCGTCAATAC3′	144	[3]

bp—base pairs.
